# Role of Exosomes in Crosstalk Between Cancer-Associated Fibroblasts and Cancer Cells

**DOI:** 10.3389/fonc.2019.00356

**Published:** 2019-05-03

**Authors:** Xi Yang, Yida Li, Liqing Zou, Zhengfei Zhu

**Affiliations:** ^1^Department of Radiation Oncology, Fudan University Shanghai Cancer Center, Fudan University, Shanghai, China; ^2^Department of Oncology, Shanghai Medical College, Fudan University, Shanghai, China

**Keywords:** cancer-associated fibroblasts, exosomes, microRNA, lncRNA, crosstalk

## Abstract

Cancer-associated fibroblasts (CAFs) are important cells of the tumor microenvironment that can communicate with tumor cells through various mechanisms. Recently, increasing studies have found that exosomes transmit biological information by carrying microRNAs, lncRNAs, proteins, metabolites, and other substances, and thus exert biological and therapeutic effects. CAF-secreted exosomes can also affect the tumor phenotype, while the exosomes released by tumor cells can activate CAFs. Here, we review the role of exosomes in the crosstalk between CAFs and tumor cells and elaborate its mechanism.

## Introduction

Fibroblasts are common cells of the connective tissue and are very crucial in the wound healing process (1). In many cases, tumors are analogous to wounds that do not heal, the reconstruction process of stromal cells shares some similarities with the wound healing process. Activated fibroblasts also exist in cancer stroma and are commonly known as cancer-associated fibroblasts (CAFs) (2).

CAFs express specific markers distinct from normal fibroblasts (NFs) and participate in tumor microenvironment construction to promote tumor invasion, proliferation, and metastasis. They can produce a variety of proinflammatory factors and even recruit other types of stromal cells to the primary and the metastatic sites of cancer (3). The multiple signaling axes, including IL-6/STAT3, FGF-2/FGFR1, NF-κB, and TGF-β1/SMAD axes appear to be abnormally activated in CAFs compared to those of NFs. The exact origin of CAFs and the mechanism of how normal cells become CAFs is still unclear, but based on current evidence, it is reasonable to believe that a considerable number of CAFs originate from NFs surrounding cancer cells (4).

Exosomes are membrane-enclosed vesicles derived from the endosomal system during the formation of multivesicular bodies, with a diameter of ~30–100 nm (5). In carcinogenesis, exosomes participate in proliferation, angiogenesis, immunosuppression, and preparation of premetastatic niches in secondary organs (6). Exosomes have been widely reported to mediate local and systemic cell communication through the horizontal transfer of information via microRNAs, long non-coding RNAs (lncRNAs), proteins, mRNAs, metabolites and other substances. Also, exosomes are considered to play an anti-tumor role by carrying and transporting various substances such as cisplatin (7, 8), paclitaxel (9), and RNA (10). However, the mechanisms of packaging and release of exosomes have not been completely characterized (11).

**Figure 1:**
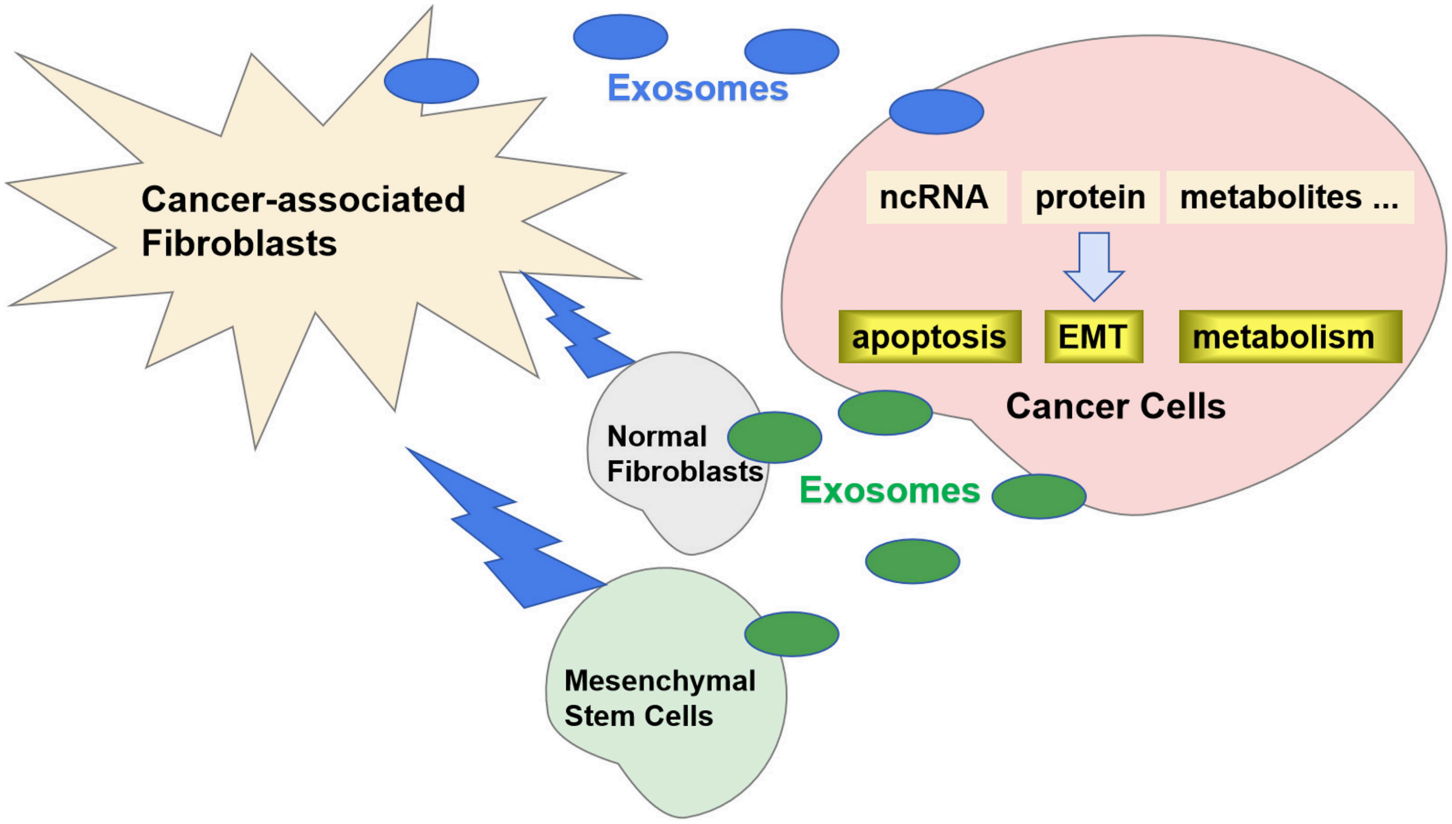
Exosomes in crosstalk between CAFs and cancer cells. Specific exosomes released from CAFs can be internalized by cancer cells and regulate carcinogenesis by transferring ncRNA, proteins, and metabolites. Correspondingly, the exosomes released by cancer cells can also promote the transformation of CAFs from NFs and MSCs.

Recently, it has been reported that exosomes play an important role in the crosstalk between CAFs and cancer cells (12). Specific exosomes released from CAFs can be internalized by cancer cells and contribute to the progression and metastasis by transferring various types of substances (Figure 1). Correspondingly, the exosomes released by cancer cells can also promote the transformation of CAFs. In this review, we will discuss the role of exosomes in the crosstalk between CAFs and cancer cells.

## Role of CAF-Derived Exosomes in Carcinogenesis

In 2012, Luga et al. first proved that CAFs in breast cancer stroma can secrete CD81-positive exosomes, which promote breast cancer cell (BCC) motility, protrusive activity, and metastasis by activating Wnt-PCP autocrine signaling in BCCs. The published gene expression data of carcinoma-associated stroma indicated that in human CAFs, CD81 expression is upregulated and might be correlated with the disease stage (13). However, in different tumor microenvironments, exosomes from CAFs may differ significantly. Miki et al. reported that CD81 is unexpressed in exosomes released from gastric cancer CAFs, and only scirrhous-type gastric cancer cells can uptake CD9-positive exosomes released from CAFs, which can promote cancer cell migration and invasion by activating the MMP2 signaling pathway (14). Hence, exosomes can play specific roles in cancer cells and these roles might be related to exosome-specific membrane proteins. The carcinogenic ability of exosomes is attributed to non-coding RNAs (ncRNA), proteins, metabolites and other substances present within them.

### Non-coding RNA (ncRNA)

Non-coding RNAs (ncRNA) are receiving considerable attention in exosome research and are modulators of recipient cells in most instances, thereby favoring tumor development (15). In many liquid biopsy sample types, large amount of miRNAs are packaged into exosomes (16). Certain types of circulating miRNAs are strongly correlated with the progression of different cancer types. Herrera et al. extracted RNA from exosomes of CAFs and NFs from colorectal cancer (CRC) tissues and normal mucosa tissues, resected from nine patients, respectively. Next-generation sequencing (NGS) results showed that the levels of 52 ncRNAs differed between exosomes derived from NFs and CAFs, and bioinformatic analysis revealed that CAF-derived exosomes can affect cancer cells and other cells in the tumor microenvironment, thereby promoting tumor progression (17).

Various miRNAs are enriched in CAF-derived exosomes and regulate cancer cells via multiple mechanisms. miR-21 has been shown to exhibit oncogenic effects across several tumor types, among which the best-described interactions are those with tumor suppressor genes, such as PTEN, p21, and PDCD4 (18). Yeung et al. by NGS analysis found that the expression of miR-21 was significantly higher in CAF-secreted exosomes than in exosomes from ovarian cancer cells. Functional studies revealed that miR-21 is exosomally transferred from CAFs to cancer cells, where it suppresses ovarian cancer cell apoptosis and increases paclitaxel resistance by binding to its novel, direct target APAF1(19). Similarly, Bhome et al. found that miR-21 is enriched and is present abundantly in CAF-derived exosomes. They used miR-21 overexpressing fibroblasts and CRC cells in orthotopic xenografts and observed increased liver metastasis compared to those established with control fibroblasts (20). Donnarumma et al. found that the expression levels of miR-21, miR-378e, and miR-143 were increased in exosomes from CAFs as compared to those from NFs in breast cancer patients. Transfection of BCCs with these three miRNAs promoted stemness and epithelial mesenchymal transition (EMT) in these cells (21).

Other miRNAs are also involved in cancer proliferation. Zhang et al. by miRNA sequencing found that miR-320a level was significantly reduced in exosomes than in matched para-cancer fibroblasts in patients with primary hepatocellular carcinoma (HCC). miR-320a can bind to PBX3, thereby suppressing proliferation, migration and metastasis of HCC cells. Xenograft experiments involving CAFs mixed with MHCC97-H cells showed that miR-320a overexpression in CAFs can suppress carcinogenesis (22). Besides, miR-34a-5p (23) and miR-148b (24) in CAF-derived exosomes regulate the EMT of oral squamous cell carcinoma (OSCC) and endometrial cancer cells, respectively. miR-196a in CAF-derived exosomes binds to novel targets, such as CDKN1B and ING5, in head and neck cancer cells, resulting in cisplatin resistance (25). CAF-derived exosomes do not always lead to malignant phenotypes in cancer cells. Li et al. found that CAF-derived exosomes could inhibit the growth, invasion and metastasis of cholangiocarcinoma tumors by carrying miR-195 *in vitro*. This observation was also confirmed in a rat model of cholangiocarcinoma (26). CAFs might also have different biological effects in different tumor microenvironments. Estrogen receptor (ER) expression in ER-positive breast cancer cells was significantly decreased after co-culturing with conditioned media from CAFs derived from ER-negative breast cancer cells. Further, studies have found that this phenomenon might be due to the release of CAF-derived exosomes from ER-negative breast cancer cells containing miR-221/222. Since miR-221/222 knockdown rescued ER repression in ER-positive breast cancer cell lines (27). Using NFs and esophageal cancer cells, Nouraee et al. developed a co-culture system that mimicked the tumor microenvironment. They detected increased expression of miR-33a and miR-326 in exosomes from co-cultured conditioned media, which indicated that miRNAs secreted from CAFs could play a role in tumor microenvironment (28).

Recently, many studies have found that exosomes secreted by tumor-associated macrophages (TAMs) and mesenchymal stem cells (MSCs) can regulate the biological behavior of tumors by carrying lncRNA (29). LncRNA might also exist in CAF-derived exosomes in addition to miRNAs. Ren et al. found that CAFs expressed significantly higher levels of lncRNA-H19 than NFs. Exosomal H19 from CAFs could promote stemness and oxaliplatin resistance in CRC cells by activating the β-catenin pathway via acting on miR-141 through competitive endogenous RNA sponge mechanism (30).

### Proteins

Proteins in CAF-derived exosomes are mostly associated with EMT in cancer cells. Chen et al. reported that p85α is an essential protein of stromal fibroblasts, and loss of p85α could stimulate fibroblasts to express and secrete additional Wnt10b, which is transported to adjacent epithelial cancer cells, thus activating the EMT pathway via Wnt/β-catenin signaling, thereby leading to BCC metastasis (31). Li et al. found that TGF-β is upregulated in CAF-derived exosomes compared to normal omentum fibroblasts in ovarian cancer patients. Further, *in vitro* experiments showed that CAF-derived exosomes were taken up by ovarian SKOV-3 and CAOV-3 cells, thereby activating the SMAD signaling pathway and enhancing the migration and invasion capacity and promoting EMT in ovarian cancer cells (32). Recently, Hu et al. found that exosomal Wnts released from CAFs induce de-differentiation of CRC cells into cancer stem cells (CSCs) and promote chemoresistance *in vitro* and *in vivo* (33).

### Other Substances

In addition to ncRNAs and proteins, the presence of other substances inside CAF exosomes, including DNA and metabolites, have been reported. Nabet et al. showed that stimulation of stromal NOTCH-MYC by BCCs leads to high RN7SL1 levels driven by an endogenous RNA POL3, which is usually shielded by SRP9/14 RNA binding protein. The elevated RN7SL1 level alters its stoichiometry with SRP9/14 thus generating unshielded RN7SL1 in stromal exosomes. Upon its transfer to BCCs, unshielded RN7SL1 acts as an activator of PRR RIG-I, thereby promoting proliferation, metastasis and resistance to treatment. Combined with evidence from patient blood and tumor tissues, these results demonstrated that the regulation of RNA unshielding connects stromal activation with the use of RNA DAMPs that promotes carcinogenesis (34). Richards et al. found that gemcitabine-treated CAFs abundantly secrete exosomes that contain chemoresistance-promoting factors, such as miR-146a and snail mRNA, which are transferred to the pancreatic ductal adenocarcinoma cells and then promoted chemoresistance and proliferation. Finally, a reduction in exosome release reduces the chemoresistance-promoting abilities of CAF cells (35).

Zhao et al. found that CAF-derived exosomes could inhibit mitochondrial oxidative phosphorylation, thereby increasing glutamine-dependent reductive carboxylation and glycolysis in cancer cells. The 13C isotope-labeling experiments showed that exosomes can provide amino acids for nutrition-deficient cancer cells through a mechanism similar to that of macro-cytoplasmic cell proliferation. They performed GC-MS and ultra-high-performance liquid chromatography (UPLC) experiments and confirmed that exosomes in both prostate and pancreatic CAFs contain complete metabolites, including amino acids such as glutamine, threonine, serine and valine; lipids such as palmitate and stearate; TCA cycle intermediates such as citrate, pyruvate, a-ketoglutarate, fumarate and malate, which are extensively used by cancer cells for carbon metabolism, in case of nutritional deficiency or nutritional stress thus promoting tumor growth (36).

## Role of Exosomes in the Transition of CAFs

CAFs arise from neighboring NFs or other cells that undergo a differentiation process induced by tumor cells and develop invasive and migratory capacities. Increasing studies have revealed that CAFs originate from various cells through different mechanisms related to exosomes, and the most common origin are NFs and MSCs (4).

### Normal Fibroblasts

Compared to NFs, CAFs overexpress markers such as α-smooth muscle actin (α-SMA), fibroblast activation protein (FAP), and galectin. Genetic heterogeneities have also been detected in CAFs (37). Array data of primary cultures of CAFs vs. paired NFs from resected breast cancer tissues identified 11 dysregulated miRNAs, and the predicted target genes were mainly related to migration, secretion, adhesion, and cell-cell interaction (38).

The abundant miRNAs in exosomes of tumor cells play an important role in reprogramming of NFs into CAFs. In breast cancer, when exosomes from cancer cells were co-cultured with NFs, an alteration of such “CAF phenotype” was observed. miR-9 was found to convert NFs into CAFs, and the overexpression of miR-9 also identified a signature of different genes related to cell motility and ECM organization such as MMP1, EFEMP1, and COL1A1 (39). In melanoma, exosomal miR-155 induces the proangiogenic switch of CAFs by inhibition of SOCS1 expression and then activates the JAK2/STAT3 signaling pathway (40). On the contrary, miR-211 in melanoma exosomes could block the switch of CAFs by activation of the IGF1R/MAPK signaling pathway (41). In addition, various novel miRNAs were investigated recently. For example, metastatic HCC cells secrete exosomal miR-1247-3p that targets B4GALT3, thereby activating β1-integrin via NF-κB signaling in fibroblasts (42). In OSCC cells, exosomes containing lncRNA-CAF released from cancer cells could increase its expression in stromal fibroblasts, which then upregulated IL-33 by blocking its degradation by p62-dependent autophagic activity, promoting the conversion of NFs into CAFs (43).

Proteins delivered by exosomes can also induce NF-CAF transition. Webber et al. found that exosomes could deliver TGF-β and promote NF differentiation into myofibroblasts (44). Exosomal TGF-β accounts for 53.4–86.3% of the total TGF-β present in the cancer cell supernatant, and exosomal TGF-β is localized inside exosomes (45). Moreover, exosomal TGF-β instead of cell-secreted TGF-β is involved in the activation of SMAD signaling, thereby inducing NF-CAF transition. Recently, Rai et al. performed proteomic profiling and functional dissection of colorectal cancer cell-derived exosomes. They found that these exosomes could activate normal quiescent fibroblasts into CAF-like fibroblasts. Interestingly, fibroblasts activated by exosomes derived from primary and metastatic cancer cells have distinct protein profiles and functions, and exhibit elevated expression of pro-angiogenic proteins (IL8, NDRG1, RAB10), pro-proliferative proteins (FFPS, SA1008) pro-invasive regulators of membrane protrusion proteins (MYO1B, PDLIM1), and matrix-remodeling proteins (MMP11, ADAM10, EMMPRIN) (46).

### Mesenchymal Stem Cells (MSCs)

MSCs also give rise to CAFs. They are fibroblast-like cells which can be isolated from different kinds of tissues such as fresh umbilical cords and adipose tissue. In gastric cancer mouse models, at least 20% of CAFs originate from the bone marrow and are derived from MSCs (47). Recent studies have shown that tumor exosomes can interact with local and distant MSCs, both in transforming MSCs and setting a premetastatic niche (48, 49). Cho et al. demonstrated that exosomes from breast and ovarian cancer cells can induce a phenotype of tumor-supporting myofibroblasts on MSCs (50, 51). Different miRNAs, such as miR-21 and miR-146a, that are known as critical regulators of CAF induction, MSC proliferation, and angiogenic activities, are involved in this process (4, 52, 53). However, further investigations are needed to understand the specific underlying mechanism. Various proteins also take part in this process although some of them are limited in the exosomes. For example, tetraspanins can be delivered into MSCs by exosomes thereby promoting activation, growth, and motility which are recognized as the characteristics of CAFs (54). Interestingly, similar to NF-CAF transition, TGF-β pathway was investigated as well, and a study verified that the TGF-β/TGF-β R1 interaction mediates Smad2/3 activation and increases the expression of FAP and α-SMA in MSCs, which is always recognized as CAFs (55). However, since there is no established definition to distinguish CAFs from MSCs, more studies are needed to elucidate the role of exosomes in the transformation between MSCs and CAFs.

CAFs are also found to originate from other cells. Endothelial cells can be induced by TGF-β and converted into CAFs through endothelial to mesenchymal transition (56). Pericytes are identified as a reservoir for CAFs induced by tumor-derived exosomes in gastric cancer, and the transition of pericytes to CAFs is induced by exosome-mediated BMP transfer and activation of PI3K/AKT and MEK/ERK pathways (57). Besides, CAFs are also thought to originate from fat cells (58).

## Prospect

Exosomes play an important role in the crosstalk between CAFs and cancer cells, thereby contributing to carcinogenesis and tumor microenvironment. With the increasing importance of tumor microenvironment in cancer treatment, the study of exosomes and its mechanism in crosstalk might be a promising direction in the future. However, the tumor microenvironment is extremely complex, and the relationship between CAFs and cancer cells is far from the simple interaction of cells *in vitro*. For example, acidic cancer environment will produce completely different biological effects compared to normal environment (59, 60). Some studies have found that both the quality and quantity of exosomes will change in an acidic environment (61, 62). These observations also indicate that the research aspects discussed in this review should be viewed in a broader context. In the future, more in-depth and innovative research is required to elucidate the role of exosomes in the crosstalk between CAFs and cancer cells.

## Author Contributions

ZZ contributed to the conception and design of the review. XY and YL wrote the manuscript. LZ revised the manuscript. All authors contributed to manuscript revision, read, and approved the submitted version.

### Conflict of Interest Statement

The authors declare that the research was conducted in the absence of any commercial or financial relationships that could be construed as a potential conflict of interest.
